# Folding analysis of the most complex Stevedore’s protein knot

**DOI:** 10.1038/srep31514

**Published:** 2016-08-16

**Authors:** Iren Wang, Szu-Yu Chen, Shang-Te Danny Hsu

**Affiliations:** 1 Institute of Biological Chemistry, Academia Sinica, 128, Section 2, Academia Road, Taipei 11529, Taiwan

## Abstract

DehI is a homodimeric haloacid dehalogenase from *Pseudomonas putida* that contains the most complex 6_1_ Stevedore’s protein knot within its folding topology. To examine how DehI attains such an intricate knotted topology we combined far-UV circular dichroism (CD), intrinsic fluorescence spectroscopy and small angle X-ray scattering (SAXS) to investigate its folding mechanism. Equilibrium unfolding of DehI by chemical denaturation indicated the presence of two highly populated folding intermediates, I and I’. While the two intermediates vary in secondary structure contents and tertiary packing according to CD and intrinsic fluorescence, respectively, their overall dimension and compactness are similar according to SAXS. Three single-tryptophan variants (W34, W53, and W196) were generated to probe non-cooperative unfolding events localized around the three fluorophores. Kinetic fluorescence measurements indicated that the transition from the intermediate I’ to the unfolded state is rate limiting. Our multiparametric folding analyses suggest that DehI unfolds through a linear folding pathway with two distinct folding intermediates by initial hydrophobic collapse followed by nucleation condensation, and that knotting precedes the formation of secondary structures.

Topologically knotted proteins have in recent years attracted tremendous attentions from biophysicists because of their abilities to spontaneously fold into natively knotted conformations despite the apparent structural complexities^1^,^2^,^3^. To date, hundreds of knotted protein structures have been identified in the database^4^,^5^,^6^,^7^, and systematic annotations of their knot types and biological functions are now made available in the KnotProt database^8^. The majority of knotted proteins contain a simple trefoil knot (3_1_)^9^,^10^,^11^,^12^, while more complex knot types, such as figure-of-eight knots (4_1_)^13^,^14^ and Gordian knots (5_2_)^2^ have been identified in proteins of higher organisms. The most complex protein knot is a Stevedore’s knot (6_1_), which was identified in the structure of DehI, an α-haloacid dehalogenase from *Pseudomonas putida* strain PP3 (Fig. 1)^15^. While biological implications of these protein knots remain to be established, it has been proposed that protein knots should be of functional importance given their presence throughout evolutions across different proteins families with little sequence similarity^16^.

The folding pathways of several knotted proteins have been investigated in detail both experimentally and computationally. The best examples are the trefoil-knotted bacterial RNA methyltransferases YibK and YbeA^17^,^18^,^19^,^20^. It has been shown that both proteins retain their knotted structures under highly denaturing conditions^21^ despite the lack of appreciable secondary structures^22^,^23^ or compactness^24^. Indeed, it has been demonstrated that both proteins can knot themselves without any auxiliary chaperone during *de novo* folding while the addition of chaperonins significantly accelerates the folding rates, which are similar to rates at which these proteins refold from chemically denatured and knotted states^25^,^26^. We have recently delineated the folding mechanism of the smallest trefoil knotted protein MJ0366 from *Methanocaldococcus jannaschii*^27^. Similar to YibK and YbeA, the unfolding rate of this small knotted protein is slow compared to the typical unfolding rates of small globular proteins of similar sizes^28^. By concatenating two identical repeats of an all-helical and homodimeric protein, HP0242 from *Helicobacter pylori*, an artificially engineered trefoil-knotted protein was created by Yeates and coworkers^29^. The knotted variant is more stable than its parent construct but folds at a remarkably slower rate, suggesting that knotting is the rate-limiting step^30^. In addition to trefoil-knotted proteins, the folding of Gordian knotted proteins, human UCH-L1 and UCH-L3, have been investigated^31^,^32^,^33^. Similar to YibK, these Gordian knotted proteins also exhibit parallel folding pathways with distinct folding intermediates that are hyperfluorescent in the case of UCH-L3^31^, and are highly populated during equilibrium unfolding in the case of UCH-L1^32^.

In contrast to ensemble spectroscopic experiments, computational analyses provide detailed mechanistic insights into the folding mechanism of knotted proteins at atomic resolution. While non-native interactions have been suggested to play a critical role in knot formation^34^,^35^,^36^, several coarse-grained simulations using structure-based model have shown that it is possible to impose native contacts alone with differential weighing factors to achieve knotting into native structures^37^,^38^,^39^,^40^. According to these simulations, slip-knotting has been put forward to be a likely mechanism for protein knotting^37^,^38^,^39^,^40^,^41^. The need to back-track from misfolded conformations, some of which are knotted into wrong handedness, may account for the slow folding rates compared to those of the unknotted counterparts, and, importantly, knotting is suggested to be the rate-limiting step^39^,^41^,^42^. Indeed, back-tracking has been observed experimentally for HP0242 variants^30^. Furthermore, untying a deep protein knot was also proven to be only possible at very high simulation temperatures^36^,^43^, which is consistent with the experimental finding that uses chemical denaturant to unfold knotted proteins without being able to untie them^44^. Depending on the knot type and depth of the knot, lattice model-based simulations have shown knotting may take place at a much later stage for a 5_2_ knot when most native contacts are formed as opposed to the concomitant formation of transition state and the knotted structure for a 3_1_ knot^45^.

Contrary to the wealth of knowledge available to other knotted proteins, there is a lack of information regarding the folding mechanism of DehI, which is the first example of the most complex Stevedore’s 6_1_ knot known to date (Fig. 1)^15^,^46^. DehI adopts a unique dehalogenase fold in contrast to the unknotted α/β fold that is common among dehalogenases^46^. DehI is homodimeric and there is a two-fold symmetry formed by two highly symmetric N- and C-terminal domains within each monomer, corresponding to residues 1–130 and 166–296, respectively. A linker (residues 131–165) forming a number of hydrogen bonds and salt-bridges in between the two domains connects them to complete the 6_1_ knotted topology (Fig. 1). This is reminiscent to the construction of an engineered protein knot by linking two unknotted subunits within a homodimeric HP0242 protein by means of gene duplication^29^,^47^. Indeed, a coarse-grained simulation on DehI suggested that the domain linker is involved in slip-knotting and loop-flipping to act as a knot-promoting loop^15^.

To gain further insights into the folding mechanism of the most complex 6_1_ knotted protein, we combined far-UV circular dichroism (CD), intrinsic fluorescence spectroscopy and small angle X-ray scattering (SAXS) to investigate the folding dynamics and kinetics of DehI. Our results revealed the presence of two highly populated folding intermediates during equilibrium unfolding by chemical denaturation. These intermediates differ in their secondary structure contents and tertiary packing, but exhibit similar degrees of structural order and compactness. Through comparison of the spectroscopic characteristics, thermodynamic and kinetic parameters of single-tryptophan variants with those of the wild type (wt), a linear folding pathway with two distinct folding intermediates was proposed for DehI.

## Results

### Equilibrium unfolding of DehI monitored by various biophysical probes

To assess the solution structure of DehI, we used size-exclusion chromatography coupled with multi-angle light scattering (SEC-MALS) to confirm the homodimeric state of DehI. The observed molecular weight (MW) corresponding to the mono-dispersed major elution peak is 67.0 ± 1.5 kDa, which is in good agreement with the theoretical MW of a homodimeric DehI as being 69.9 kDa (Figure S1A). The far-UV CD spectrum indicates that DehI is highly helical with two well-defined peak signals at 208 and 222 nm with a predicted α-helical content of 54.9%, which is consistent with the crystal structure of DehI that is 59.5% α-helical (Figure S1B). Using the reported crystal structure of DehI as input, the theoretical SAXS profile can be back-calculated^48^, and a good agreement with the experimental SAXS profile was obtained, indicating that DehI adopts the same tertiary structure in solution as that observed in crystallized state (Figure S1C).

Having established the solution structure assembly of DehI, we next examined the equilibrium unfolding of DehI induced by guanidine hydrochloride (GdnHCl) using far-UV CD and intrinsic fluorescence spectroscopy to monitor the changes in secondary and tertiary structures, respectively (Fig. 2). The far-UV CD spectra display monotonously decreased signals on increasing GdnHCl concentration (Fig. 2A). In contrast, the intrinsic fluorescence spectra exhibit rapid increase in intensity with a red shift 15 nm from 320 nm to 335 nm between 0 and 1 M GdnHCl, followed by signal reduction and a further red shift to 350 nm (Fig. 2B). Singular value decomposition (SVD) analysis^30^,^49^ of the resulting isotherms reveals a four-state equilibrium unfolding behavior with two highly populated folding intermediate states (I and I’). The transition from the native state (N) to the first intermediate state (I) occurs at *ca.* 1 M GdnHCl; the transition from I to the second intermediate state (I’) occurs at *ca.* 2 M GdnHCl; the transition from I’ to the fully denatured state (U) occurs at *ca.* 3.5 M GdnHCl (Table 1).

We next employed SAXS to monitor the global conformational changes in DehI as a function of GdnHCl concentration. The resulting Kratky plots–the observed SAXS intensity, I, multiplied by the square of momentum transfer, q, *i.e.,* I*q^2^, as a function of momentum transfer, q–report on the degree of order and disorder of DehI^24^,^50^ as a function of GdnHCl concentration. A peak distribution indicates the presence of a highly ordered structure whereas the presence of monotonously increased signals reflects the presence of highly disordered structure, *i.e.*, unfolding (Fig. 2C). SVD analysis of the Kratky plots reveals three distinct populations with the two transition points between the three states coinciding with the N–I and I’–U transitions observed by far-UV CD and intrinsic fluorescence (Table 1). Note that analysis of the far-UV CD spectra of I and I’ indicates that the former retains 42% of the native helical structure while the latter retains only 21%. Therefore, the multi-parametric analyses indicate that the two folding intermediates have distinct secondary structure contents while their degrees of disorder (or compactness) are indistinguishable to SAXS.

### Characterization of single-tryptophan variants of DehI

DehI has three endogenous tryptophan residues, namely W34, W53 and W196, that are strategically located within the three dimensional structure: W53 is located at the N-terminal domain, W34 is located at the hinge between the two domains, and W196 is located at the C-terminal domain (Figure S2A). By generating single-tryptophan variants of which two tryptophan residues are mutated into phenylalanine, the remaining tryptophan can inform us on the localized structural changes around the only tryptophan. The far-UV CD spectra of the three single-tryptophan variants are comparable with that of wt, indicating that the two tryptophan-to-phenylalanine mutations result in minimal structural perturbations to DehI (Figure S2B). Although the replacements of tryptophans by phenylalanine result in marginally reduced thermal stabilities in the variants–the melting temperatures of W34, W53, and W196 are 58.9 ± 0.0, 63.2 ± 0.3, and 61.6 ± 0.3 °C, respectively, compared to 66.3 ± 0.6 °C of wt (Figure S3)–the dehalogenase activities of the single-tryptophan variants are essentially identical to that of wt (Figure S4).

Close examination of the spectral characteristics of the intrinsic fluorescence spectra of the single-tryptophan variants indicate that all endogenous tryptophan residues are buried in non-polar environments as evidenced by their blue-shifted maximum emission wavelength (λ_max_) to less than 330 nm (Fig. 3)^51^. Among the three variants, W53 exhibits a λ_max_ (325 nm) that is closer to that of wt while those of W34 and W196 (310 nm) are significantly blue-shifted. The red-shifted fluorescence of W53 can be rationalized by the presence of two charged side-chains, D25 and K148, in close proximity to W53, which form salt bridges between each other, while W34 and W196 have no apparent charged groups in their neighborhood (Figure S5).

### Equilibrium unfolding of DehI variants by intrinsic fluorescence spectroscopy

We next evaluated the equilibrium unfolding of the three single-tryptophan variants in comparison with wt using intrinsic fluorescence and far-UV CD spectroscopy (Figs 3 and S6, and Table 1). Both spectroscopic probes reveal wt-like four-state unfolding characteristics for the single-tryptophan variants. Nevertheless, intrinsic fluorescence was more informative in the SVD analysis; we therefore focus on the fluorescence-based findings in the following. The primary difference between W34 and wt is the large *m*-value associated with the N–I transition (*m*_N–I_ = 9.71 ± 1.08 kcal·mol^−1^M^−1^) of W34 that is 50% larger than that of wt. The large *m*-value suggests major changes in solvent accessible surface area during the N–I transition, which is attributed to dimer dissociation, supported by the non-linear concentration dependence in fluorescence change in the presence of 1 M GdnHCl whereas the intrinsic fluorescence intensity is linearly proportional to protein concentration at all other GdnHCl concentrations examined (Fig. 4A). Since W34 is buried within the hydrophobic core in between the symmetric N- and C-terminal domains as a hinge (Figure S2), the larger *m*_N–I_-value may reflect significant conformational rearrangements between the two domains upon dimer dissociation thereby resulting in changes in the structure/folding of the hinge linker.

Furthermore, W53 and wt share a similar *m*-value associated with the I–I’ transition (*m*_I–I’_ = 5.58 ± 0.40 and 5.30 ± 0.30 kcal·mol^−1^M^−1^, respectively), while the other two variants, W34 and W196, exhibit much smaller *m*-values (0.99 ± 0.16 versus 1.38 ± 0.14 kcal·mol^−1^M^−1^, respectively) suggesting that the structural elements around W53 remain native-like in the I state and become solvent exposed only during the I–I’ transition. Compared to W34 and W53, the structural elements around W196 become largely disordered/unfolded after the N–I transition, hence little spectroscopic change is monitored in the following I–I’ and I’–U transitions, while the characteristics of the I’–U transitions of W34 and W53 are similar to that of wt (Fig. 3 and Table 1). The equilibrium unfolding results indicate that the equilibrium unfolding process of DehI is not fully cooperative, and that each of the three variants probes distinct unfolding events during the four-state unfolding process.

### Kinetics of DehI and variants by stopped-flow fluorescence measurements

To further investigate the mechanism of the equilibrium intermediates in the kinetic folding of DehI, we carried out the analysis of the DehI variants unfolding using stopped-flow and manual mixing fluorescence measurements. Note however, that the following kinetic measurements are limited to the unfolding parts given that the equilibrium unfolding of DehI is not fully reversible during the N_2_–2I transition around 1M GdnHCl (Fig. 4B,C), while folding reversibility could be established for folding events in the presence of higher concentrations of GdnHCl (>1M). As expected from equilibrium unfolding results (Fig. 2B), the intrinsic fluorescence of wt increases monotonously as a function of unfolding time after rapid mixing with GdnHCl (Fig. 5). The kinetics of unfolding is mainly biphasic for wt between 1.5 and 3.7 M GdnHCl. This covers the transitions between the two intermediates as observed in equilibrium unfolding. We noticed, however, that a small but significant kinetic phase was present when folding took place between 2.5 and 3.0 M GdnHCl (crosses in Fig. 5A), suggesting the presence of structurally heterogeneous intermediates under these conditions. Beyond 3.7 M GdnHCl, single exponential fitting is sufficient to account for the observed unfolding kinetic traces for wt corresponding to the I’-U transition, which is rate limiting. In the cases of W34 and W53, we could observe multiple unfolding kinetic phases corresponding to where the two equilibrium intermediates become populated as does wt. In particular, an additional hyperfluorescent folding intermediate^52^ is transiently populated during unfolding above 3 M GdnHCl (open squares in Fig. 5A and kinetic traces in Fig. 5B). In contrast to the multiphasic unfolding kinetics of W34 and W53, the unfolding kinetics of W196 is monophasic throughout the GdnHCl concentration range used herein, and no apparent intermediate was found compared to other variants, indicating a simpler unfolding process within the micro-environment of W196.

Extrapolations of the observed unfolding rates of wt to zero molar denaturant yield three unfolding rates, 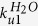 = 6.3 × 10^−7^ s^−1^, 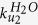 = 9 × 10^−3^ s^−1^, and 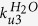 = 1.4 s^−1^, spanning six orders of magnitudes and corresponding to the large, intermediate and small kinetic *m*-values (5.3, 2.2, and 0.8 kcal·mol^−1^·M^−1^, respectively; see Table 2). The slowest unfolding rates, *k*_*u1*_, of the three variants are comparable with similar kinetic *m*-values compared to wt, while W53 contains an extra phase to give a 10-fold faster *k*_*u1’*_ (~4.7 × 10^−6^ s^−1^; triangle symbols fit with a solid line in blue, Fig. 5A) than *k*_*u1*_. W34 exhibits a 30-fold faster *k*_*u2*_ with a comparable kinetic *m*-value of 1.6 kcal·mol^−1^·M^−1^ compared to wt, while the unfolding kinetic phase appears in higher amounts of denaturant (>2.5 M GdnHCl; solid circles in Fig. 5A), which is absent in W53 and W196 (Fig. 5A and Table 2). W34 reveals a set of comparable values of *k*_*u3*_and *m*_*ku3*_ as wt (open squares in Fig. 5A and Table 2), suggesting that W34 is responsible for this kinetic phase observed in wt. For W53, in addition to the tentatively assigned *k*_*u3*_ (filled circles in Fig. 5A) that is shared with wt, there is an additional unfolding kinetic phase that is slower in rates (open squares in Fig. 5A), suggesting the presence of distinct folding intermediates that were probed specifically by W53. W196 reveals a much simpler monophasic unfolding and the observed *k*_*u3*_ becomes slightly faster than other variants. In all cases, the intercept of the multi-phasic unfolding rates coincide with the I’–U transition points, implying that the I’–U transition is rate limiting.

## Discussion

**Figure 1:**
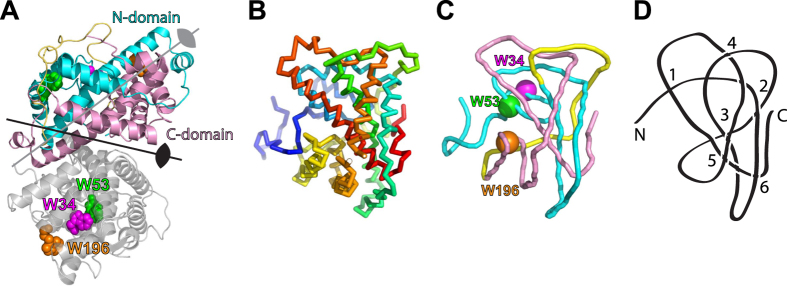
Structure and knot topology for DehI. (**A**) Cartoon representation of the crystal structure of DehI (PDB code: 3BJX). The N- and C-terminal domains of one subunit are colored in cyan and pink, respectively, and the connecting linker in yellow. The other subunit is colored in grey with the three tryptophan residues labeled with their respective sequence numbers. The two-fold symmetry axes of the homodimer and two symmetric domains are indicated by arrows. (**B**) Simplified backbone representation of a DehI monomer. The ribbon diagram is color-ramped from blue to red from the N- to C-termini. (**C**) Reduced backbone topology of DehI generated by the pKNOT web server using the Taylor smoothing algorithm^7^. The positions of the three tryptophan residues are indicated. (**D**) The simplified 6_1_ knot topology of DehI with the positions of the six crossings indicated.

**Figure 2:**
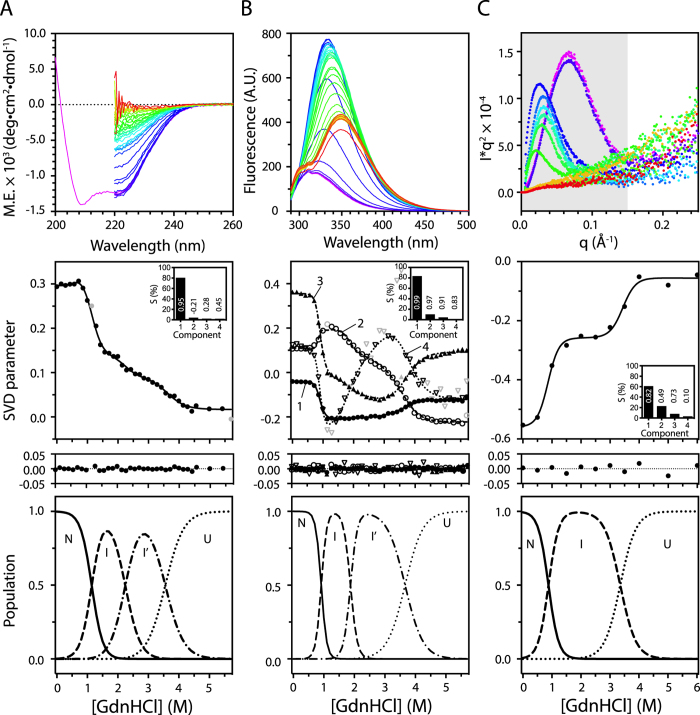
GdnHCl-induced equilibrium unfolding of DehI monitored by (**A**) Far-UV CD, (**B**) intrinsic fluorescence and (**C**) SAXS. Top panels: GdnHCl titration series of far-UV CD and fluorescence emission spectra of DehI recorded from 0 to 5.7 M GdnHCl with a linear gradient with equal spacing, colored ramped from purple to red. Kratky plots of the SAXS data collected in the presence of 0, 0.5, 1, 1.5, 2, 2.5, 3, 3.5, 4, 5, and 6M GdnHCl. Middle panels: SVD analysis outputs of far-UV CD, intrinsic fluorescence and SAXS data. Insets: the percentage and auto-correlation coefficients individual components. Residuals of data fitting are shown below. Bottom panels: the population of native state (solid line), intermediate state (dashed line) and unfolded state (dotted line) for DehI as a function of GdnHCl concentration. Populations of individual states are calculated using the thermodynamic parameters obtained from the fitting to a three-state or four-state folding model.

**Figure 3:**
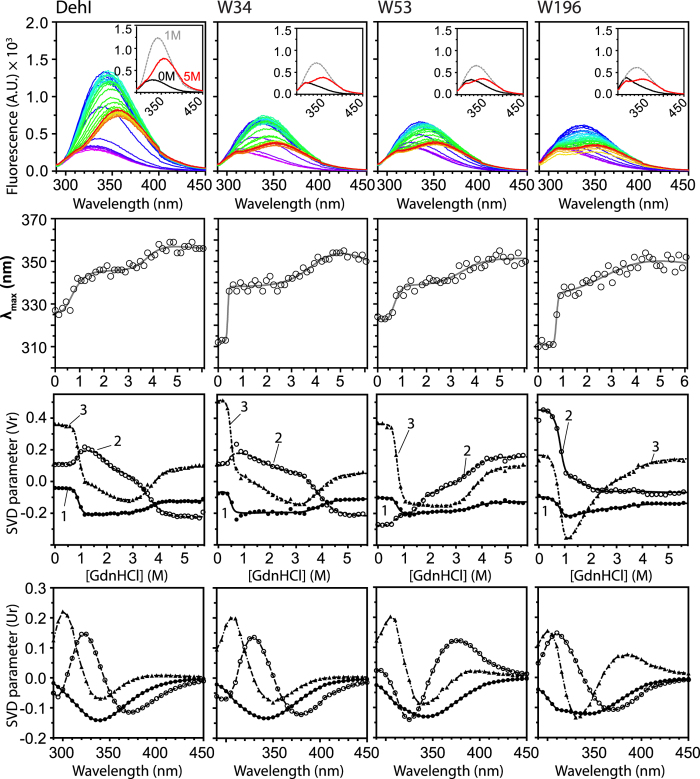
Comparison of the folding equilibria of DehI variants. The fluorescence emission spectra, maximum emission wavelength (λ_max_), SVD components V_r_ (as a function of GdnHCl concentration) and U_r_ (as a function of wavelength) are shown in descending order for wt, W34, W53 and W196 from left to right. The fluorescence emission spectra recorded from 0 to 6 M GdnHCl are ramped from purple to red. Insets: Three selected spectra in the presence of 0, 1, and 5M GdnHCl colored in black, grey and red, respectively. Significant SVD components V_r_ are global-fit to a four-state equilibrium unfolding model to extract associated thermodynamics parameters (Table 1).

**Figure 4:**
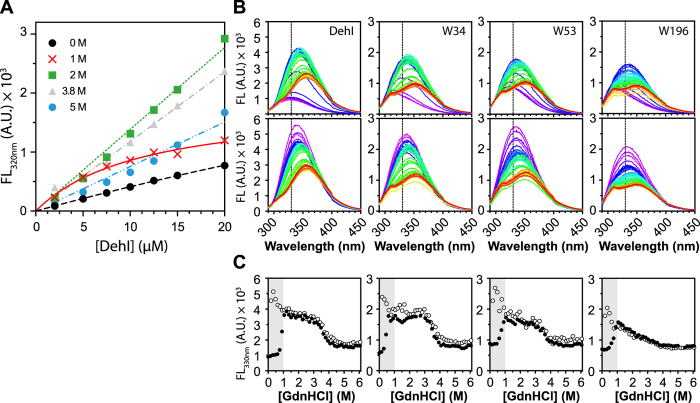
The dimerization formation of DehI is irreversible. (**A**) Assessment of dimer dissociation by protein concentration-dependent intrinsic fluorescence. Protein concentration dependence in fluorescence change (emission wavelength 320 nm) was measured under various GdnHCl concentrations. (**B**) Equilibrium unfolding and refolding of DehI and variants. For unfolding analysis, 3 μM of native DehI and variants were incubated overnight at 0 to 6 M GdnHCl with a linear gradient with equal spacing (41 points). For refolding analysis, 100 μM 6 M GdnHCl-denatured DehI and variants were diluted into a final protein concentration of 3 μM within 0 to 6 M GdnHCl (41 points) and incubated overnight to refold. The emission fluorescence spectra were recorded using a fluorescence microplate reader. Each dataset was colored ramped from purple to red, representing as 0 to 6 M GdnHCl, as described in Fig. 2. An emission wavelength of 330 nm was indicated with a dashed line. (**C**) The fluorescence changes of the emission wavelength 330 nm for DehI and variants were plotted as a function of GdnHCl concentration, where solid circles represent for the unfolding fluorescence intensity and the open circles represent for those of refolding events. The inconsistent unfolding and refolding profiles were highlighted and revealed in a grey area with the range of 0 to 1 M GdnHCl that corresponds to the transition point of dimer dissociation.

DehI contains the most complex protein knot that has been identified so far, and studying the folding mechanism of a complex homodimeric, all-helical structure of DehI by itself presents a major challenge. Here we report the detailed multiparametric experimental characterization on the folding equilibrium and kinetics of DehI. According to the equilibrium unfolding results, we propose that a native dimeric DehI (N_2_) dissociates into a monomeric intermediate (2I) at the first transition point as evidenced by the non-linearity of the intrinsic fluorescence as a function of protein concentration (Fig. 4A). The intermediate I retains significant amount of secondary structure within the N-terminal domain (see definition in Fig. 1) where W53 resides as reflected in the large equilibrium *m*_*I–I’*_ value. In contrast, the *m*_*I–I’*_ values of W34 or W196 are markedly smaller, indicating that the C-terminal domain is already unfolded prior to the I-I’ transition. Together, the N_2_-2I transition results in a loss of helical content of 58% (Fig. 2A). The monomeric intermediate (2I) subsequently converts into another intermediate (2I’) with much less secondary structure content (21% of native state) as W53 shows the largest *m*-value associated within this transition. Despite the further loss of secondary structure content, the global compactness of I’ is comparable to that of I according to the peak distribution in Kratky plot derived from SAXS measurements (Fig. 2C). Finally, the long-range interactions within I’ are lost upon unfolding into a denature state U, a random coil-like ensemble, according to its R_g_ value^24^. Taken together, we propose a linear folding pathway of DehI as illustrated in Fig. 6.

**Figure 5:**
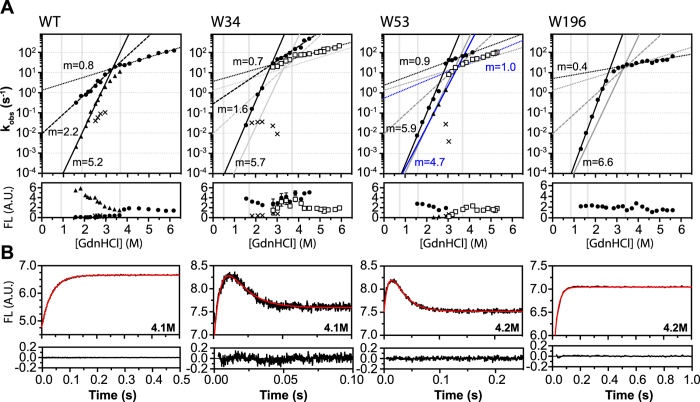
Unfolding kinetics of DehI and the three single-tryptophan variants. (**A**) GdnHCl dependence of the observed rates (k_obs_) of DehI, W34, W53 and W196. The corresponding amplitudes are shown in the lower panels, respectively, and the vertical grey lines represent for the transition points for each variants according to the chemical unfolding equilibrium data (see Table 1). The observed reaction rates with increased amplitudes are indicated in solid symbols, while those with decreased amplitudes are in open symbols. The slopes of observed unfolding rates (*m*_ku_) are listed aside the fitting lines, and the fitting curves of DehI are plotted as references for comparison. The fitting lines of the extra phases of W53 compared to the wt and W34 are colored in blue. (**B**) Selected unfolding kinetic traces of each variant (black line) reveal the altered fluorescence intensity increased or decreased before reaching the plateau. The fitting curves by single or double exponential kinetics equations are shown in red lines, and the corresponding residuals are plotted below each panel.

**Figure 6:**
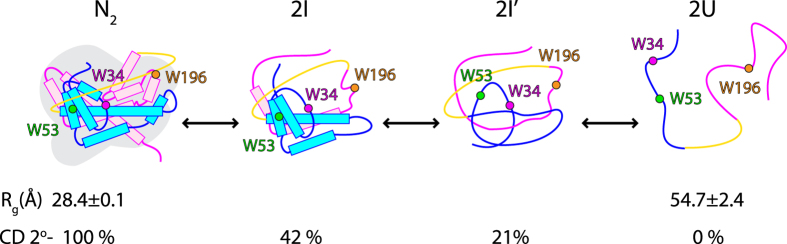
Proposed folding pathway of DehI. Schematic representation of a dimeric DehI (N_2_) is shown using the same coloring scheme as in Fig. 1, and its radius of gyration (R_g_ = 28 Å) and relative secondary structure content according to far-UV CD are indicated below. DehI first unfolds in to a monomeric intermediate state (I) that retain 42% of the secondary structure and further unfolds into another intermediate state (I’) with only 21% of the native secondary structure content but the overall compaction is similar to that of I, according to SAXS-derived Kratky plot. The final unfolding, and potentially unknotting, is achieved, resulting a total loss of secondary structure and expansion of the overall dimension to an R_g_ value of 55 Å.

Based on the reversibility of folding equilibrium of monomeric DehI between the two intermediates and denatured states (Fig. 4), we suggest the unfolded DehI first collapses into an intermediate I’ with partial secondary structure contents, and then converts into another intermediate I with a comparable compactness as I’ but accompanied with more secondary structure formations (Fig. 6). With the aid of the three single-tryptophan variants, we observed distinct sequential and non-cooperative folding events. All variants exhibit steep increases of the unfolding rates on increasing GdnHCl concentration, which are slowed down at the third transition point that is attributed to the I’-U transition, suggesting that this step is rate-limiting and that it may be associated with knot formation due to a higher kinetic barrier that needs to be overcome during folding. Once the intermediate I’ is formed, significant compaction takes place resulting in a well-defined peak distribution in the Kratky plot (Fig. 2C). When I’ converts into the intermediate I, more secondary structures are formed, and W53 is sequestered while the remaining parts are still solvent assessable, implying that DehI folds with a hierarchic folding process reminiscent to a nucleation condensation model^53^. The final step of native dimer formation appears to be highly frustrated and aggregation-prone as reflected by the irreversibility of the equilibrium refolding at this stage (Fig. 4), suggesting the necessity of auxiliary chaperones to achieve efficient folding *in vivo*. While our linear folding pathway can account for most of our experimental finding, additional structural heterogeneity does exist under intermediate conditions, as reflected by the presence of multiphasic unfolding kinetics in the presence of intermediate GdnHCl concentrations (Fig. 5). For example, an additional hyper-fluorescent phase during the I’-U transition in W34 and W53 was observed (open squares in Fig. 5). This may be attributed to non-specific collapse after the effective loop flipping to form a knot.

Virnau and co-worker have carried out coarse-grained simulations using a structure-based model to propose that the folding of DehI starts by forming two long loops, namely S- and B-loops, followed by flipping of one over the other to form an intertwined, twisted conformation, and is completed by flipping of the end of a slipknot to attain the native, knotted conformation^15^. However, whether such sequential events indeed occur as proposed remains to be established experimentally. Recent experimental findings by using a trefoil-knotted methyltransferase from *Thermotoga maritima* suggested that knot formation could be decoupled from folding^54^. Both of the computational and experimental work proposed a flipping route for knotting formation with a flip of a knot-crossing loop that leads to untying or tying. In this regard, the rate-limiting step of DehI (I’-U transition) is consistent with the observation that the knotting formation occurs under the loops flipping by long-range interactions while less secondary structures (local folding events) are generated during this process.

In conclusion, we report the folding mechanism of DehI using a multiparametric biophysical approach. The use of the three single-tryptophan variants is pivotal for dissecting the involvements of sub-domains in the proposed folding mechanism. The equilibrium unfolding analyses of DehI variants yielded consistent results that a native dimeric DehI (N_2_) dissociates into a partially disordered monomeric intermediate, I, followed by further unfolding of secondary structures into a distinct intermediate, I’, while the degree of compactness is retained to be comparable to that of I. Finally complete unfolding results in a random coil-like unfolded state, 2U, with negligible secondary structure content. The results provide experimental basis for further understanding of the folding mechanism of knotted protein.

## Material and Methods

### Sample preparation

The construct of DehI in a pET15b vector was a kind gift from Dr. Matthew Wilce at Monash University, Australia^46^. It was transformed into *Escherichia coli* BL21 (DE3) for recombinant protein production in LB (Luria-Bertani) broth at 37 °C in the presence of ampicillin (100 μg/ml) for antibiotics selection. Recombinant protein over-expression was induced by adding a final concentration of 0.5 mM IPTG (isopropyl β-D-1-thiogalactopyranoside) when the cell density reached an optical density at 600 nm (OD_600_) of 0.6–0.8, and the cell culture was further incubated for 4 h at the same temperature. After harvesting the cells by centrifugation, the cell pellet was resuspended by buffer A (20 mM potassium phosphate (pH 8) and 300 mM NaCl) with 100 μg/ml lysozyme and DNase I. The cells were lysed by sonication and the supernatant with soluble DehI was separated from cell debris by centrifugation at 4 °C and 45,000 × g for 30 min. The supernatant was directly applied to a 5-ml HisTrap HP column (GE Healthcare Life Sciences), washed by buffer A with 20 mM imidazole to remove non-specifically bound proteins. Recombinant DehI was eluted by buffer A with 120 mM imidazole. The eluted fraction was further purified by size exclusion chromatography using a Hiload 26/60 Superdex 200 column (GE Healthcare Life Sciences) to homogeneity (>95%) by visual inspection of the Coomassie-blue-stained sodium dodecyl sulfate (SDS) polyacrylamide gel (PAGE). The purified protein was concentrated to 100 μM within buffer A, aliquotized, flash-frozen by liquid nitrogen and stored at −30 °C until further use. All biophysical analyses were performed in buffer A unless stated otherwise.

### Far-UV circular dichroism (CD)

Aliquots of 4 μM DehI variants in buffer A were incubated with a gradient of concentrations (0–6 M) of guanidine hydrochloride (GdnHCl) overnight before CD measurements as described previously^27^,^30^ using a quartz cuvette with a path-length of 0.1 cm. The CD spectra were recorded over 200–260 nm for the native protein, and 220–260 nm for the GdnHCl-denatured samples at 25 °C with a bandwidth of 1 nm, a data interval of 0.5 nm, and an averaging time of one second using a J-815 CD spectrometer (JASCO, Tokyo, Japan).

### Fluorescence-based thermal shift assay

The thermal stabilities of DehI variants were measured by a fluorescence-based thermal shift assay using LightCycler 480 instrument (Roche Life Science). Each reaction contained 15 μl total volume with 10 μM protein and 1x Protein Thermal Shift Dye in buffer A. The fluorescence change due to the dye binding to unfolded protein was monitored in the temperature ranging from temperature 20 to 95 °C.

### Intrinsic fluorescence spectroscopy

Fluorescence spectra were collected using the same samples that were used for far-UV CD measurements as described above using a FP-8500 spectrofluorometer (JASCO, Tokyo, Japan). The intrinsic fluorescence was recorded over 290–500 nm with excitation at 280 nm, a data interval of 1 nm, a response time of 0.1 second, and slit widths of 2.5 nm for both excitation and emission. The fluorescence spectra of single-tryptophan variants were recorded using the Infinite M1000 PRO plate reader (TECAN) at 25 °C over 290–450 nm with excitation at 280 nm, a data interval of 1 nm and emission and excitation slits set at 5 nm and 2.5–5 nm.

### Small angle X-ray scattering (SAXS)

Aliquots of DehI (2 mg/ml) in buffer A with 5 mM DTT and various concentrations of GdnHCl (0–6M) were used for SAXS measurements at 15 °C. The SAXS data were collected at the SAXS beamline 23A1, National Synchrotron Radiation Research Center (NSRRC) in Hsinchu, Taiwan, as described previously^24^,^55^. Six frames with 50 s exposure time per frame were recorded for each sample using an X-ray wavelength of 1.03 Å. Measurements were performed in batch mode where samples were loaded into a cell with Kapton (polyimide) windows and recorded by rocking the sample slightly to minimize radiation damage. Frames revealing radiation damage, if any, were discarded prior to data analysis. Radiation damage was check for each sample by Coomassie-blue-stained SDS-PAGE after SAXS measurements.

### Dehalogenase activity assay

The buffer used for the colorimetric dehalogenase assay was modified from the previous report^56^, consisting of 50 mM HEPES (pH 8.2), 20 mM Na_2_SO_4_ and 1 mM EDTA. The pH indicator, phenol red, was added to a final concentration of 10–20 μg/ml, and 2-chloropropionic acid was used as the substrate of DehI and dissolved in HEPES buffer to a final concentration of 10 mM. The final enzyme concentration was 10 μM. For a control experiment, buffer A was used instead of DehI stock solution. The reactions were incubated at room temperature overnight, followed by optical absorbance measurements as the buffer color changed from pink to light yellow when its pH value decreased due to proton release upon dehalogenation. The absorbance at 559 nm was measured using a JASCO V-630 UV-Vis spectrometer at 25 °C to quantify the relative enzyme activities of DehI and its variants.

### Stopped-flow fluorescence measurements

Kinetic unfolding experiments of DehI variants were recorded using a SX18 stopped flow spectrometer in fluorescence detection mode (Applied Photophysics, Leatherhead, UK). Changes in total fluorescence of the unfolding reaction were monitored using an excitation wavelength of 280 nm with a cutoff filter of 320 nm. For unfolding, 20 μM of native proteins in buffer A were mixed, at a ratio of 1:10, with buffer A containing various concentrations of GdnHCl. The system was kept at 25 °C by a circulation water bath. For kinetic measurements on longer timescales, unfolding was triggered by manual mixing, and monitored by using a J-815 CD spectrometer (JASCO, Tokyo, Japan) or an Infinite M1000 PRO fluorimeter in a 96-well microplate format (TECAN, Switzerland) for intrinsic fluorescence measurements.

### Data analysis of equilibrium and kinetics unfolding

All equilibrium unfolding data were subjected to singular value decomposition (SVD) analysis using MATLAB (MATLAB and Statistics Toolbox Release 2012b, The MathWorks, Inc., Natick, Massachusetts, United States) to determine the number of states associated with the equilibrium unfolding. The titration series of far-UV CD, intrinsic fluorescence and the integral area of the peaks derived from the Kratky plot were used to generate an m × n matrix, M, as inputs for SVD analysis, of which m corresponds to the number of recorded wavelengths and n corresponds to the number of titration points as described previously^30^. To judge the number of significant components, the normalized correlation coefficients of individual singular values were calculated and a minimum threshold of 0.8 was used for filtering. All equilibrium unfolding data were subjected to a three-state or four-state equilibrium-unfolding model as described previously^27^,^57^ based on the number of significant SVD components, and the results were tabulated in Table 1.

The observed rate constants of DehI were extracted by fitting the unfolding kinetic traces to a single, double or triple exponential function with an offset using the software package GraphPad Prism (GraphPad Software, La Jolla, California, USA). The choice of model, i.e., single, double or triple kinetic phases, was decided using the F-test statistics by Prism. The observed rate constants in the stopped-flow fluorescence measurements were further fit to a simple linear unfolding arm to extract the associated kinetic parameters as 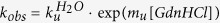, where 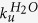 is the unfolding rate in the water, i.e., in the absence of denaturant and *m*_*u*_ is the kinetics *m*-value associated with unfolding.

## Additional Information

**How to cite this article**: Wang, I. *et al*. Folding analysis of the most complex Stevedore’s protein knot. *Sci. Rep.* **6**, 31514; doi: 10.1038/srep31514 (2016).

## Supplementary Material

Supplementary Information

## Figures and Tables

**Table 1:** Thermodynamic parameters for the fit of equilibrium unfolding data of DehI and variants.

DehI	*wt*	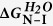 (kcal mol^−1^)	*m*_*N–I*_(kcal mol^−1^ M^−1^)	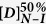 (M)	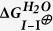 (kcal mol^–1^)	*m*_*I–I’*_(kcal mol^−1^ M^−1^)	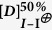 (M)	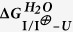 (kcal mol^−1^)	*m*_*I/I′*__*–U*_(kcal mol^−1^ M^−1^)	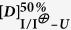 (M)
*SAXS*		2.75 ± 1.10	3.18 ± 1.23	0.87 ± 0.08				9.27 ± 5.52	2.70 ± 1.60	3.44 ± 0.16
*Far-UV CD*	*wt*	3.71 ± 1.31	3.24 ± 1.11	1.15 ± 0.10	5.40 ± 2.91	2.40 ± 1.28	2.25 ± 0.16	7.02 ± 1.20	1.98 ± 0.33	3.55 ± 0.10
	*W34*	3.10 ± 0.64	8.27 ± 1.63	0.37 ± 0.02	4.38 ± 0.67	2.53 ± 0.34	1.31 ± 0.15	4.87 ± 0.57	1.31 ± 0.15	3.71 ± 0.06
	*W53*	5.57 ± 0.95	8.57 ± 1.43	0.65 ± 0.02	5.55 ± 0.05	3.00 ± 0.51	1.85 ± 0.09	5.68 ± 0.02	1.48 ± 0.24	3.85 ± 0.08
	*W196*	5.36 ± 0.03	7.21 ± 0.73	0.74 ± 0.05	6.10 ± 0.09	3.74 ± 0.90	1.63 ± 0.10	4.51 ± 0.01	1.28 ± 0.17	3.54 ± 0.07
*Fluorescence*	*wt*	6.04 ± 0.33	6.60 ± 0.36	0.91 ± 0.01	9.92 ± 0.58	5.30 ± 0.30	1.87 ± 0.03	7.40 ± 0.32	2.02 ± 0.09	3.66 ± 0.02
	*W34*	4.83 ± 0.54	9.71 ± 1.08	0.50 ± 0.01	1.69 ± 0.08	0.99 ± 0.16	1.69 ± 0.08	6.49 ± 0.38	1.75 ± 0.10	3.70 ± 0.04
	*W53*	4.91 ± 0.34	6.74 ± 0.46	0.73 ± 0.01	8.88 ± 0.66	5.58 ± 0.40	1.59 ± 0.03	6.31 ± 0.41	1.72 ± 0.11	3.68 ± 0.05
	*W196*	5.55 ± 0.60	6.54 ± 0.70	0.85 ± 0.01	2.27 ± 0.24	1.38 ± 0.14	1.64 ± 0.06	4.21 ± 0.37	1.24 ± 0.10	3.41 ± 0.13

All experiments were recorded under the same buffer condition.

**Table 2:** Kinetic parameters for the fit of DehI and variants unfolding kinetics at pH 8 and 25 °C.

	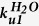 (s^−1^)	*m*_*ku1*_(kcal mol^−1^ M^−1^)	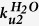 (s^−1^)	*m*_*ku2*_(kcal mol^−1^ M^−1^)	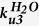 (s^−1^)	*m*_*ku3*_(kcal mol^−1^ M^−1^)
wt	(6.3 ± 2.6) × 10^−7^	5.17 ± 0.18	0.009 ± 0.003	2.23 ± 0.15	1.35 ± 0.31	0.75 ± 0.04
W34	(4.5 ± 4.0) × 10^−6^	5.68 ± 0.46	0.281 ± 0.139	1.64 ± 0.13	3.71 ± 0.86	0.69 ± 0.05
W53	(8.0 ± 6.6) × 10^−7^	5.86 ± 0.37			2.38 ± 0.71	0.91 ± 0.09
	(1.7 ± 1.1) × 10^−6^	4.69 ± 0.24			0.55 ± 0.16	1.02 ± 0.07
W196	(3.2 ± 1.1) × 10^−7^	6.61 ± 0.17			5.11 ± 1.41	0.43 ± 0.06
