# Supplementary material for: Folding analysis of the most complex Stevedore’s protein knot
Source: Sci Rep. 2016 Aug 16;6:31514. doi: 10.1038/srep31514 (PMC4985754; doi:10.1038/srep31514)
Supplement: Supplementary Information [file srep31514-s1.pdf]

Supplementary Information

Folding analysis of the most complex Stevedore's protein knot

Iren Wang[1], Szu-Yu Chen[1], Shang-Te Danny Hsu[1*]

[1] Institute of Biological Chemistry, Academia Sinica, 128, Section 2, Academia Road, Taipei 11529, Taiwan

* Corresponding author: sthsu@gate.sinica.edu.tw

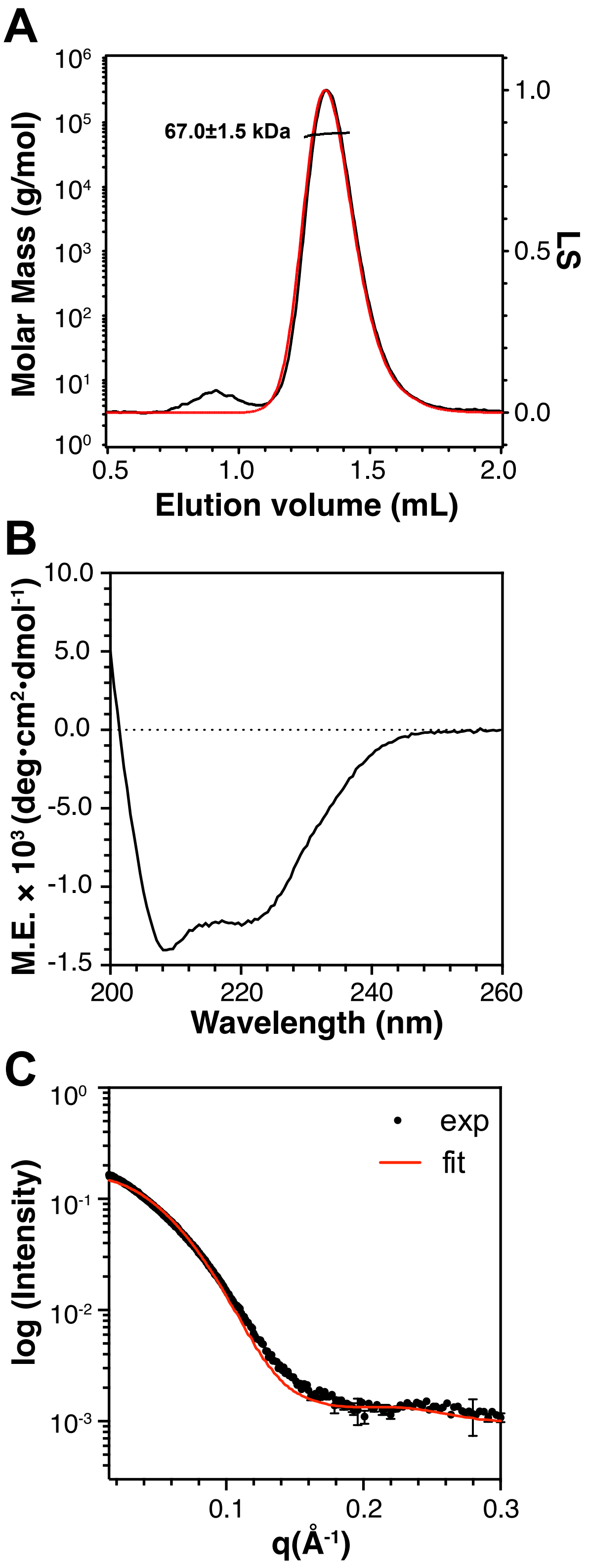

Figure S1. Evaluation of solution structure of DehI

(A) SEC-MALS analysis of DehI. The thin line segments represent for the calculated molar mass in the left axis, and the normalized UV absorbance (280 nm) in red and the normalized light scattering intensity in black are compared in the right axis. (B) Far-UV CD spectrum of DehI. (C) The solution scattering data of DehI in black dots and the evaluated SAXS profile derived from the crystal structure (PDB code: 3BJX) by CRYSOL shown in red are compared, indicating that the solution structure of DehI matches to the reported crystal structure.

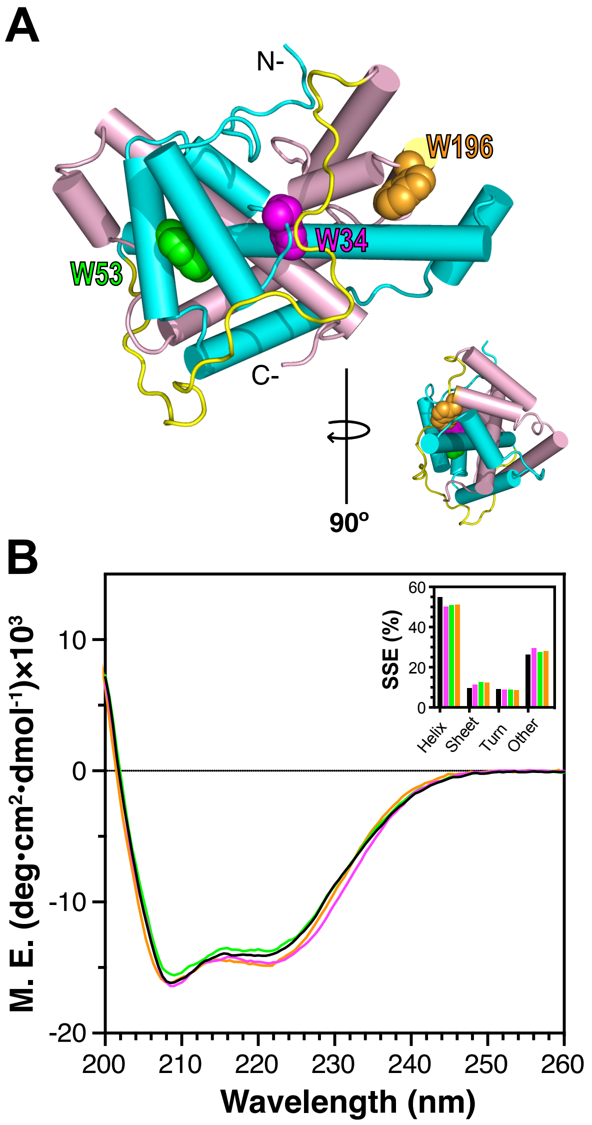

Figure S2. Structural integrity is maintained in single-tryptophan variants of DehI

(A) The two symmetric N-domain and C-domain of monomeric DehI are colored in cyan and pink, respectively, connecting with a knot-promoting loop in yellow. The three intrinsic tryptophan residues are shown in spheres. (B) Overlay of the far-UV CD spectra of wt and the three single-trytophan variants of DehI shows virtually identical spectral characteristics and similar secondary structure contents (inset).

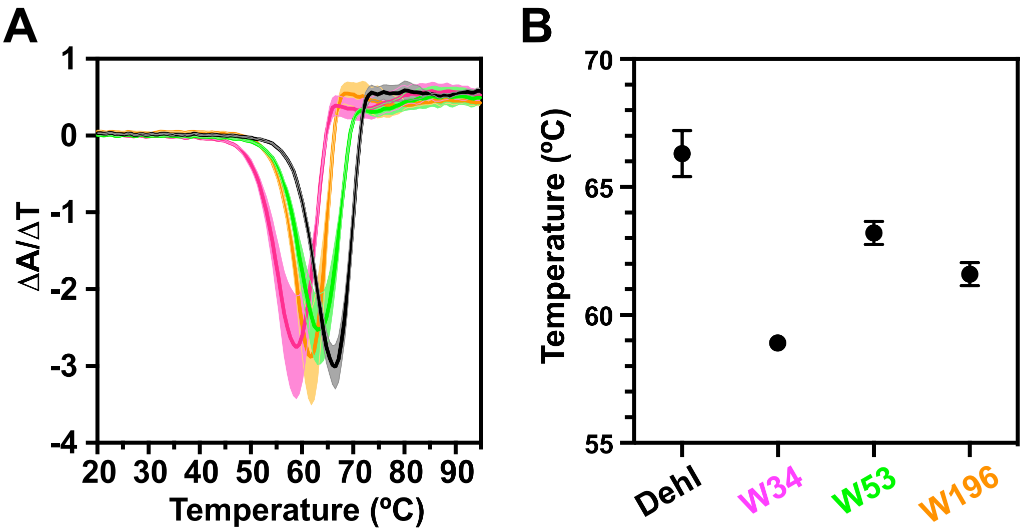

Figure S3. Thermal stability of DehI and variants

(A) The first derivative of the melting curves of DehI and its variants monitored by a fluorescence-based thermal shift assay. Wt (grey) is more thermal stable than the three variants (W34, W53 and W196 are colored in magenta, green and orange, respectively). Four independent measurements were carried out and the average values are shown in solid lines and the standard deviations are shown in shed areas. (B) The melting temperatures of DehI variants correspond to the minimum values of the first derivatives shown in (A). The error bars corresponds to the standard deviations derived from four independent measurements.

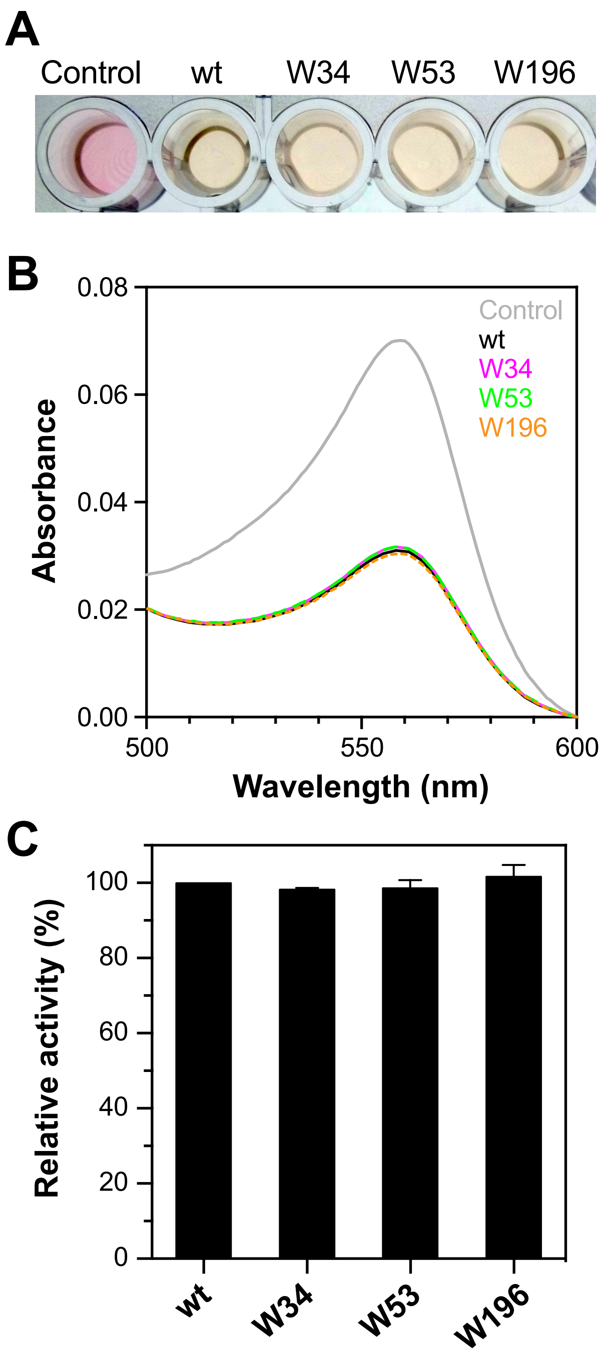

Figure S4. Haloacid dehalogenase activity of DehI and variants

(A) The enzyme activity assay of DehI and variants in microplate format reveal that all the three variants possess dehalogenase activity as wt, while the control represents the original color of phenol red by adding the protein buffer only. (B) The UV-Vis spectra of 2-chloropropionic acid in the presence of phenol red mixed with buffer A (control) and 10 μM of DehI variants. The reduction of phenol red absorbance is indistinguishable among all DehI variants, indicating that the dehalogenase activity is not altered by the replacements of tryptophan with phenylalanine. (C) The relative enzyme activity of DehI variants derived from changes in absorbance at 560 nm in (B). The activity of wt is defined as 100 %. The average values and standard deviations were derived from three independent measurements.

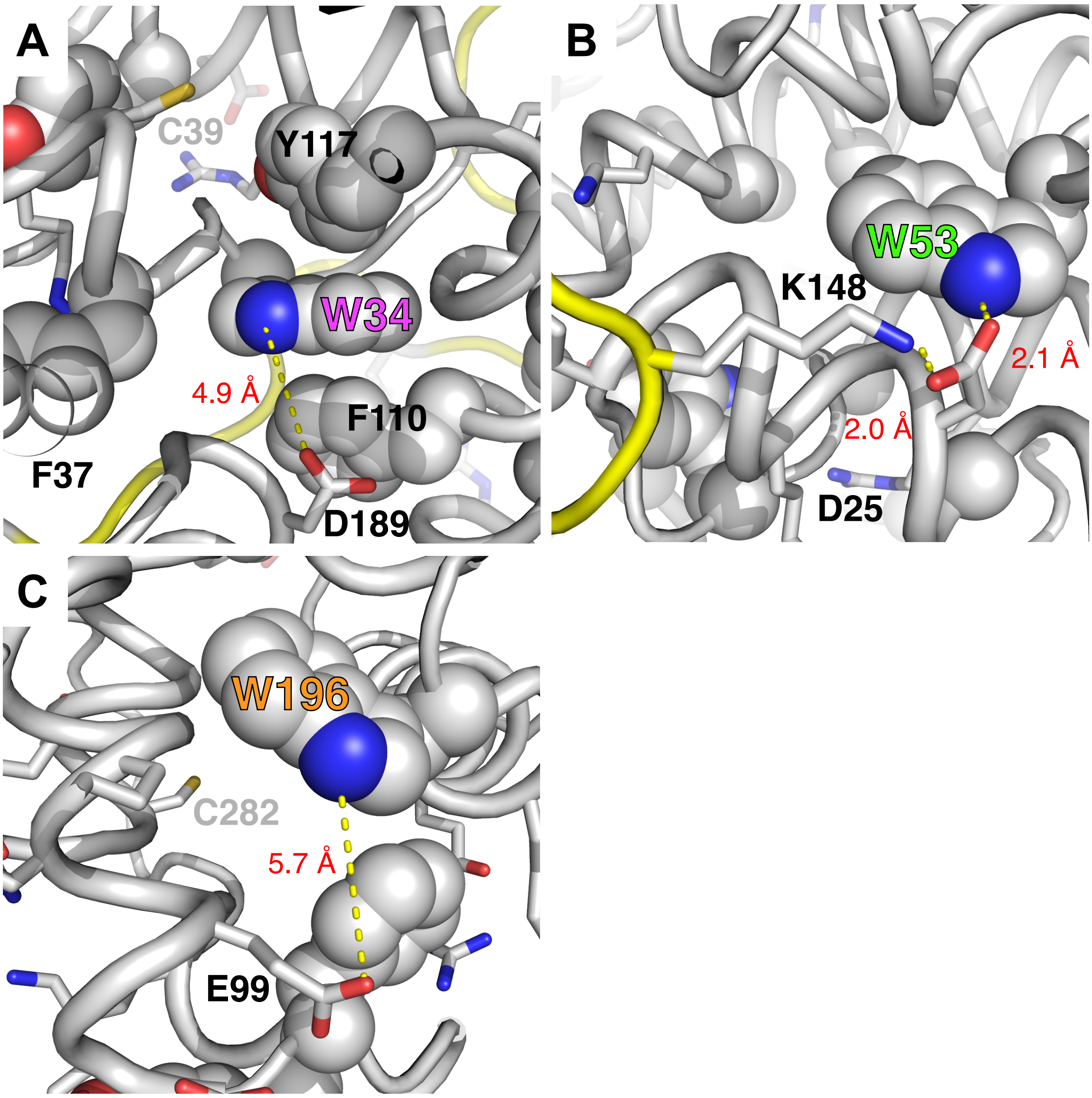

Figure S5. Structural comparison of the microenvironments of individual tryptophan residues. Charged residues in close proximity to the three endogenous tryptophan residues are shown. W53 is involved in salt bridge formation with D25 and K148. The aromatic side chains are shown in spheres, and the distances between charged atoms and the tryptophan side chain indole nitrogen are indicated in dashed yellow lines. The large knot-promoting loop is colored in yellow, and the positively charged atoms are colored in blue while negatively charged ones are in red.

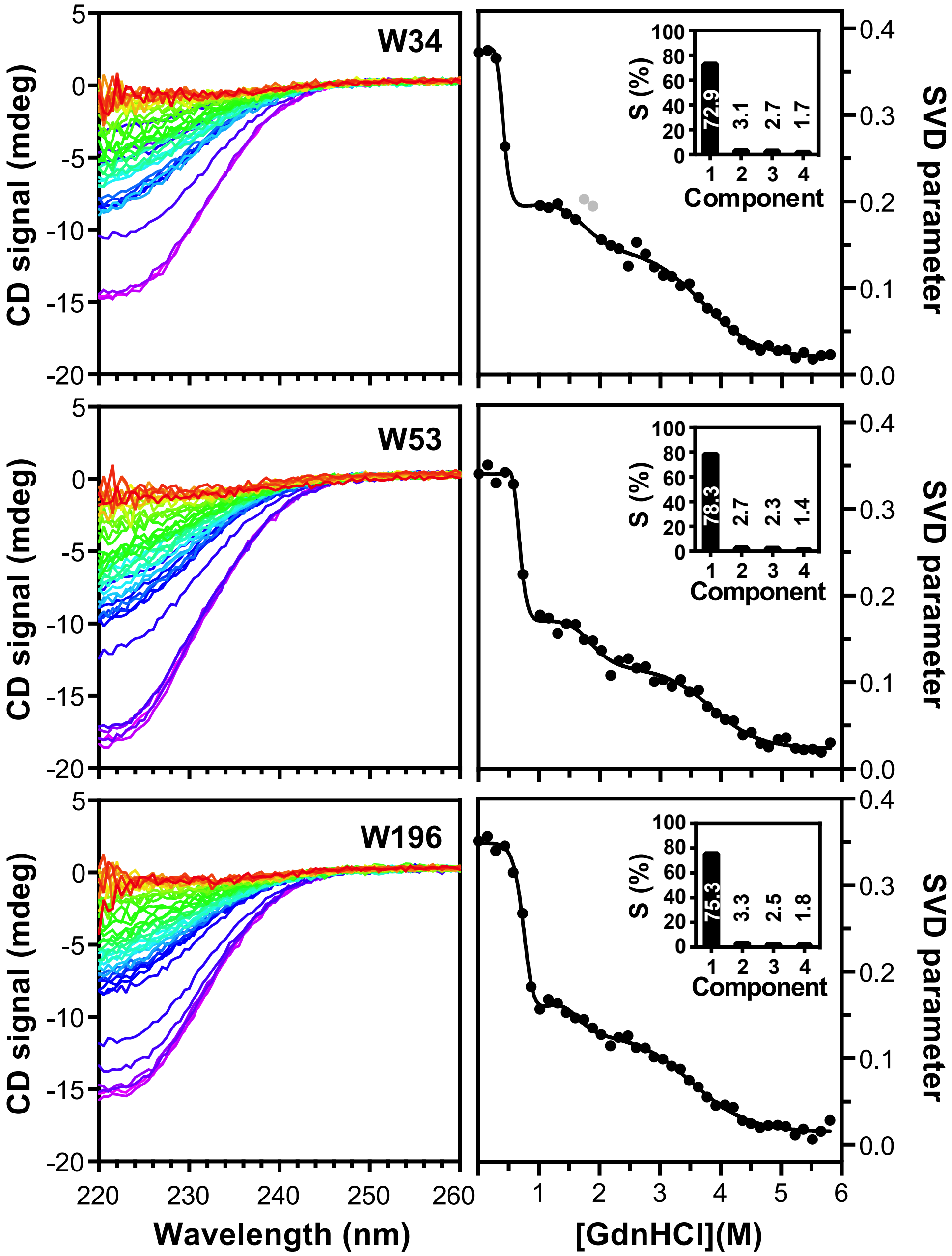

Figure S6. GdnHCl-induced equilibrium unfolding of the three variants by far-UV CD

Left: GdnHCl titration series of W34, W53, and W196 recorded from 0 to 5.8 M GdnHCl with a linear gradient with equal spacing, colored ramped from purple to red. Right: SVD analysis of far-UV CD data (in black dots) were fit to a four-state unfolding model (in black lines) as applied for wt, and the outlier points were colored in grey. The percentage and auto-correlation coefficients of individual components are shown in inset.
